# Postmortem MRI reveals distinct structural features in sudden unexpected death in epilepsy

**DOI:** 10.1002/epi4.70276

**Published:** 2026-05-08

**Authors:** Andrea Hill, Fenglai Xiao, Maria Thom, David Maudgil, Hannah Bergman, John S. Duncan, Owen J. Arthurs, Josemir W. Sander, Beate Diehl, Matthias J. Koepp

**Affiliations:** ^1^ Department of Clinical and Experimental Epilepsy UCL Queen Square Institute of Neurology London UK; ^2^ Chalfont Centre for Epilepsy Chalfont St Peter UK; ^3^ NIHR Great Ormond Street Biomedical Research Centre London UK; ^4^ Department of Radiology Wexham Park Hospital Slough UK; ^5^ Radiology Department Great Ormond Street Hospital London UK; ^6^ UCL GOS Institute of Child Health London UK

**Keywords:** biomarker, MRI, post‐mortem, SUDEP

## Abstract

Magnetic resonance imaging (MRI) abnormalities have been reported in individuals who later die from sudden unexpected death in epilepsy (SUDEP), but their specificity and predictive value remain uncertain. Postmortem MRI (PM‐MRI) offers a unique opportunity to distinguish structural features associated with SUDEP from changes related to epilepsy, comorbid illness, or the postmortem interval. We performed PM‐MRI in nine individuals: five with suspected SUDEP, two with epilepsy who died from non‐seizure‐related causes, and two without epilepsy who died from sudden cardiac death. Hippocampal, amygdala, and subcortical volumes were quantified using validated segmentation methods and compared with 70 healthy in vivo controls. Compared with non‐SUDEP cases, SUDEP cases showed significantly larger hippocampal (*p* = 0.014) and amygdala (*p* = 0.023) volumes, with most exceeding the healthy control mean, whereas non‐SUDEP cases consistently demonstrated volume reductions. These findings parallel in vivo MRI observations in individuals at high risk of SUDEP and are consistent with transient peri‐ or postictal structural changes. In the context of recent large‐scale studies showing few validated clinical SUDEP biomarkers beyond frequent generalized tonic–clonic seizures and sleeping alone, PM‐MRI may provide an objective approach to positively identifying individuals who died from SUDEP.

**Plain Language Summary:**

We used magnetic resonance imaging (MRI) scans taken after death to study brain changes in people who died from sudden unexpected death in epilepsy (SUDEP). We found that certain brain areas involved in seizure control and automatic body functions, such as breathing and heart rate, were larger in people who died from SUDEP than in people who died from other causes. These changes were similar to those previously seen on brain scans of people with epilepsy who are known to be at higher risk of SUDEP while they were still alive. Our findings suggest that postmortem MRI may help identify brain changes linked to SUDEP.

**Key points**

- SUDEP is a major cause of epilepsy‐related death, yet validated biological risk markers remain scarce.
- Postmortem MRI showed relative hippocampal and amygdala volume preservation or enlargement in SUDEP cases.
- These structural findings complement clinical risk factors, mainly frequent GTCS and sleeping alone.
- PM‐MRI may help identify structural signatures linked to peri‐ or postictal processes in SUDEP.

## 1 INTRODUCTION

Sudden death is estimated to be more than 20‐fold more frequent in individuals with epilepsy than in the general population, with sudden unexpected death in epilepsy (SUDEP) representing the leading epilepsy‐related cause of premature mortality.^1^ SUDEP is defined as a sudden, unexpected, non‐traumatic death in a person with epilepsy, with no toxicological or structural cause identified at postmortem examination, and parallels definitions applied to sudden cardiac death and sudden unexpected death in infancy.^2^

Neuroimaging studies increasingly suggest that SUDEP risk may be associated with structural and functional vulnerabilities in brain regions critical for autonomic, respiratory, and arousal regulation. Reported abnormalities in individuals who later died or were at high risk of SUDEP include alterations in hippocampal and amygdala volumes, thalamic changes, and abnormalities within brainstem networks involved in respiratory control.^3^, ^4^, ^5^, ^6^ More recently, in vivo studies have demonstrated increased gray matter volumes in limbic and subcortical regions in both adults and children at elevated SUDEP risk, suggesting that volume enlargement, rather than atrophy, may characterize vulnerable individuals.^6^, ^7^ These findings have been complemented by emerging electrophysiological biomarkers, including post‐ictal EEG signatures associated with impaired autonomic recovery.^8^

However, most imaging studies to date have examined individuals while alive, often months to years before death, limiting inference about peri‐terminal processes.^3^, ^6^ Postmortem neuroimaging data remain scarce, with only isolated ex vivo or regionally focused studies linking imaging findings to autonomic and respiratory regulatory regions.^9^ As a result, it remains unclear whether reported structural abnormalities represent chronic epilepsy‐related changes, transient seizure‐related effects, or imaging correlates specific to SUDEP.

Beyond frequent generalized tonic–clonic seizures (GTCS) and sleeping alone, few validated clinical or biological biomarkers for SUDEP currently exist.^10^, ^11^ Postmortem magnetic resonance imaging (PM‐MRI) offers a unique opportunity to capture brain structural signatures close to the time of death, potentially distinguishing seizure‐related peri‐ictal changes from features associated with non‐SUDEP deaths.^12^ We therefore conducted a pilot PM‐MRI study comparing individuals with suspected SUDEP to non‐SUDEP controls, including epilepsy and non‐epilepsy cases, to explore whether PM‐MRI can identify structural patterns relevant to SUDEP risk.

## 2 METHODS

### 2.1 Study cohort and case classification

At the instruction of coroners during the COVID‐19 pandemic, whole‐body PM‐MRI scans were obtained from nine individuals between 1 and 14 days after death. Seven individuals had refractory epilepsy with frequent GTCS, and two individuals had no history of epilepsy and died from sudden cardiac death. Among the epilepsy cases, five deaths were classified as suspected SUDEP and two as non‐SUDEP deaths due to bronchopneumonia, based on available clinical, circumstantial, and postmortem information. Full aerosol‐generating neuropathological autopsies were not performed; in one case, based on the PM‐MRI finding, a selective PM examination of the heart was undertaken.

Clinical characteristics, including epilepsy type, seizure frequency, medication, age, sex, and circumstances of death, are summarized in Table 1.

**TABLE 1:** Demographics.

	SUDEP	Non‐SUDEP	Stats	*p*
Age (median, IQR) (years)	50.0, 42.0	60.0, 29.0	Wilcoxon 22.000	0.556
Sex (F/M, *n*)	¼	3/1	Fisher's exact 2.723	0.206
Postmortem delay (median, IQR), (days)	6.0, 7.0	4.5, 8.0	Wilcoxon 15.5	0.286
Cause of death (etiology)	SUDEP focal epilepsy (4), IGE (1)	Bronchopneumonia (focal epilepsy, RE) 2 Sudden cardiac death		
Frequency of GTCS >3/year	5/5	2/2		
Nocturnal seizures	4/5	2/2		

Abbreviations: F, female; GTCS, generalized tonic–clonic seizures; IGE, idiopathic generalized epilepsy; SUDEP, sudden unexpected death in epilepsy; M, male; RE, Rasmussen's encephalitis.

### 2.2 PM‐MRI acquisition

**FIGURE 1:**
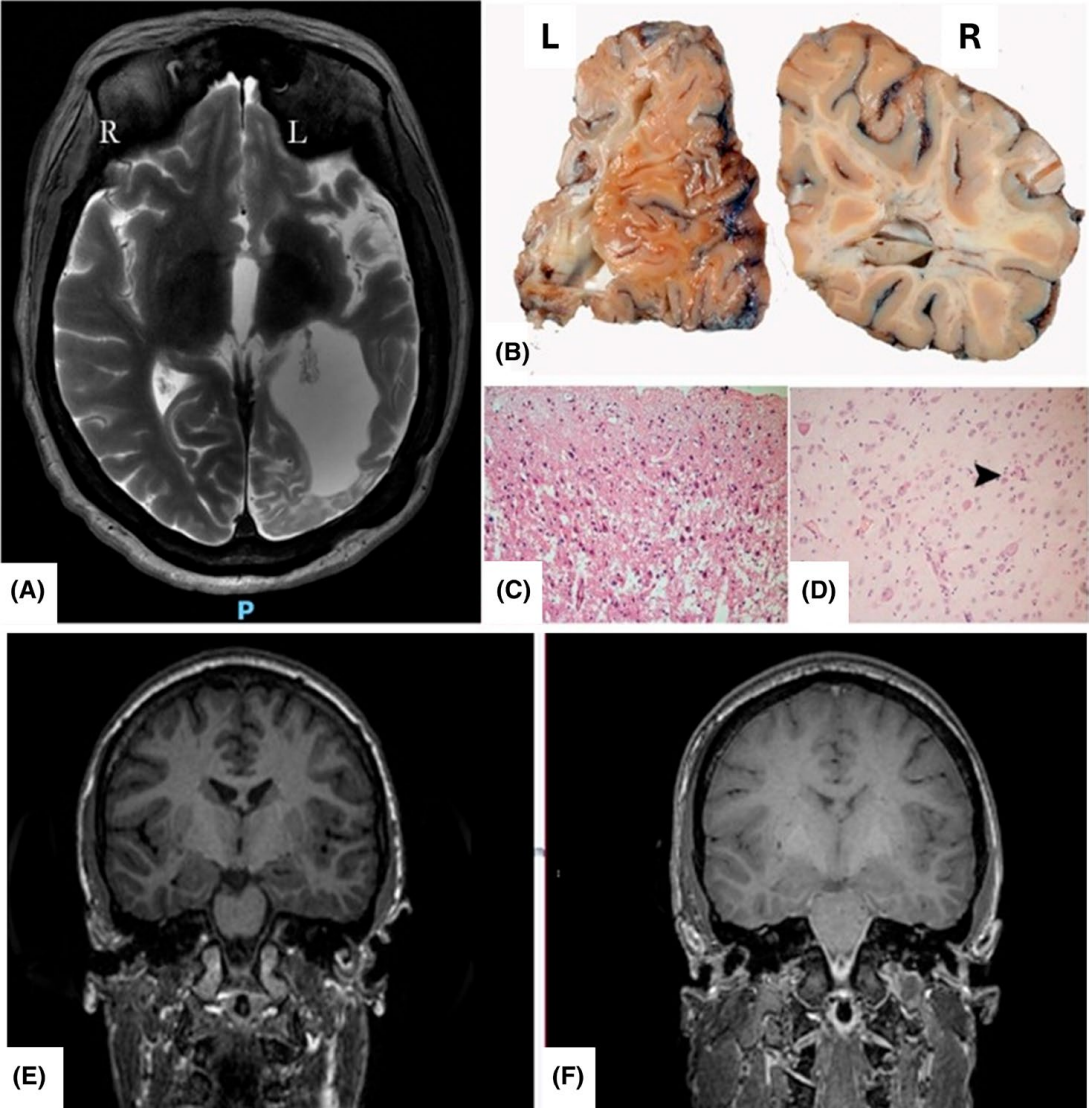
Panel Top: Rasmussen's encephalitis (RE), cause‐of‐death: pneumonia. Frequent FBTCS (~10/month) and status epilepticus (~25 during life‐time). Left: PM‐MRI; Upper right: Pathology‐confirmed left atrophy; Lower right: Histology of late stage RE in contrast to active inflammation on biopsy of same RE patient 26 years previously. Panel bottom: SUDEP (postmortem interval 7 days). Left: Axial T2 pre‐(in vivo) MRI; Right: Axial T2 postmortem.

Imaging was performed on a 3T GE Discovery MR750 scanner using an epilepsy‐specific protocol. Sequences included a 3D T1‐weighted MPRAGE acquisition (TE/TR/TI: 3.1/7.4/400 ms; voxel size: 1.0 × 1.0 × 1.0 mm). The brain was imaged in situ as part of a whole‐body PM‐MRI examination. For illustrative purposes, Figure 1 displays representative pre‐mortem and postmortem images from the same individual, acquired at comparable orientations.

### 2.3 Volumetric analysis

Volumes of the hippocampus, amygdala, and subcortical structures (thalamus, caudate, putamen, and globus pallidus) were quantified. These regions were selected because they are well circumscribed on automated segmentation and include regions, which have been implicated in prior SUDEP and autonomic regulation studies.^3^, ^4^, ^5^, ^6^, ^7^ Brainstem nuclei were not analyzed due to the limited reliability of automated volumetry in small postmortem cohorts.

Hippocampal volumes were derived using Hipposeg, a validated multi‐atlas segmentation method.^13^ Subcortical volumes were extracted using the Geodesic Information Flows (GIFs) parcellation framework.^14^ All volumes were corrected for total intracranial volume (TIV). Given the small sample size, left and right hemispheric volumes were treated separately and pooled for analysis to maximize statistical power.

Reference values were obtained from 70 healthy individuals scanned in vivo using the same imaging protocol. In vivo reference data were applied uniformly across SUDEP and non‐SUDEP groups to enable relative comparisons, acknowledging the limitations of using in vivo data as reference for postmortem measurements.

### 2.4 Statistical analysis

Group differences between suspected SUDEP and non‐SUDEP cases were assessed using Mann–Whitney *U* tests with Bonferroni correction for multiple comparisons. Comparisons with healthy controls were descriptive, with statistical testing reported where appropriate. Statistical analyses were performed using SPSS version 29 (IBM).

### 2.5 Ethics

Written informed consent for correlation with postmortem imaging was obtained from all families. The study was classified by the institutional review board as a service evaluation involving anonymized analysis of previously acquired data; therefore, individual consent for secondary analysis was not required.

## 3 RESULTS

### 3.1 Cohort characteristics

Of the seven individuals with epilepsy, five deaths were classified as probable SUDEP and two as non‐SUDEP deaths due to bronchopneumonia. All epilepsy cases had frequent GTCS. Nocturnal seizures were reported in four of the five SUDEP cases and in both non‐SUDEP epilepsy cases, placing six of seven epilepsy patients in a high‐risk SUDEP category based on established clinical risk factors.^10^, ^11^ Two additional individuals without epilepsy died from sudden cardiac death and served as non‐neurological postmortem controls (Table 1).

### 3.2 Volumetric findings

**FIGURE 2:**
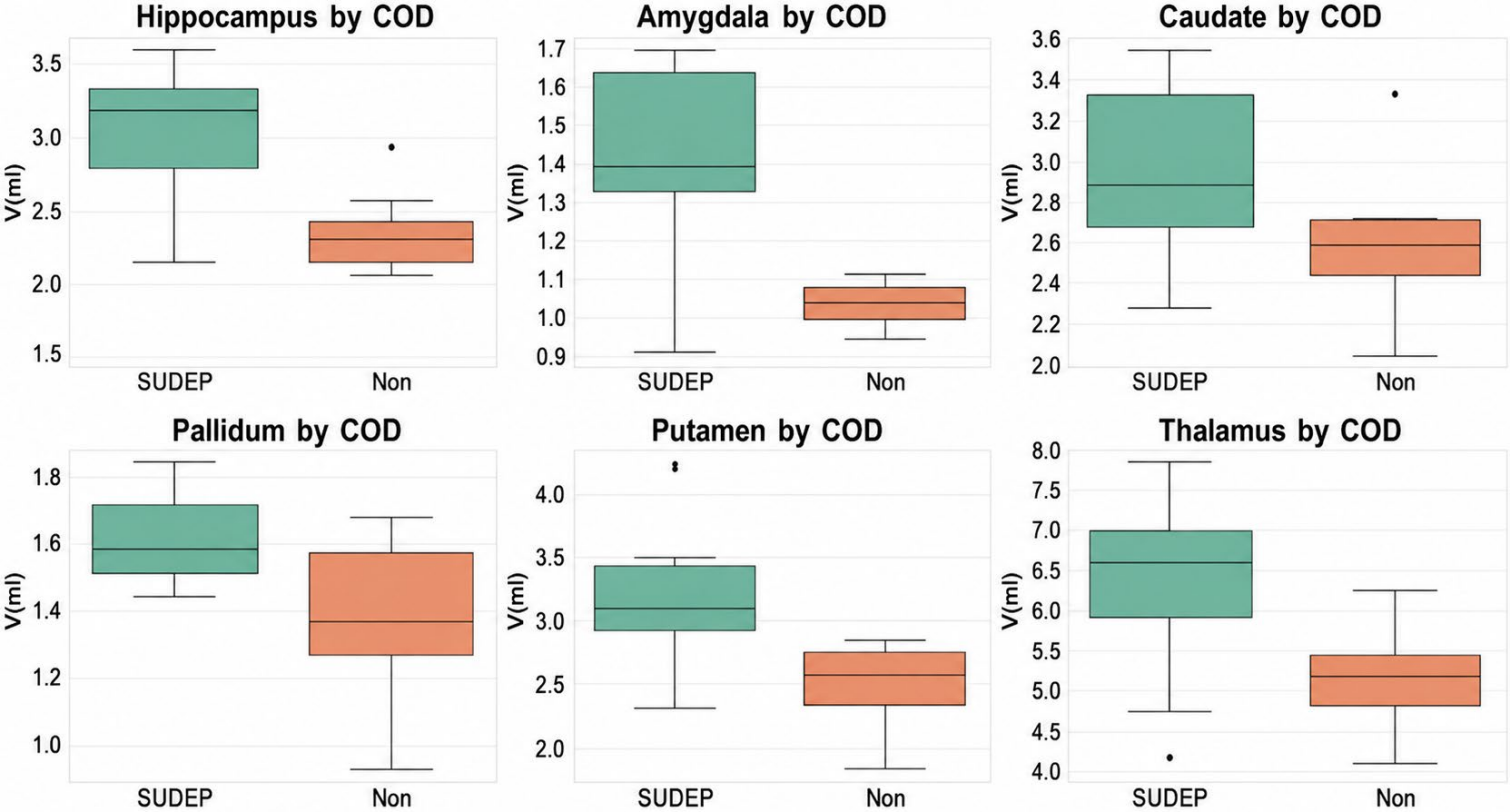
Volumetric findings: Hippocampal/amygdala and subcortical volumes (ml) in five people with epilepsy who died from SUDEP (green) and four people with epilepsy who died from non‐seizure or epilepsy‐related cause of death (red).

Compared with non‐SUDEP cases, probable SUDEP cases demonstrated larger volumes across all examined regions, including the hippocampus, amygdala, thalamus, putamen, and globus pallidus (Figure 2). Statistically significant differences after correction were observed for hippocampal and amygdala volumes, while differences in other subcortical structures showed consistent directional trends.

Relative to healthy in vivo controls, four of five SUDEP cases exhibited enlarged hippocampal and amygdala volumes. In contrast, all non‐SUDEP cases, both epilepsy‐related and non‐epilepsy deaths, demonstrated reduced volumes relative to the control mean, with amygdala volumes up to 48% below reference values.

These findings indicate divergent postmortem volumetric patterns between SUDEP and non‐SUDEP deaths, despite comparable epilepsy severity among epilepsy cases.

## 4 DISCUSSION

Our PM MRI findings demonstrate relative preservation or enlargement of hippocampal, amygdala, and subcortical volumes in individuals who died from probably SUDEP compared with non‐SUDEP controls, despite comparable epilepsy severity and seizure burden. Importantly, these findings closely mirror in vivo MRI observations reported in individuals at high risk of SUDEP and in those who later died suddenly, particularly in the context of frequent nocturnal generalized tonic–clonic seizures.^3^, ^4^, ^5^, ^6^ More recently, Roy et al. reported increased gray matter volumes in limbic and subcortical regions in children at risk of SUDEP, providing independent confirmation that volumetric enlargement, rather than atrophy, can characterize SUDEP vulnerability in vivo.^7^ The convergence between ante‐mortem and postmortem imaging strengthens the biological plausibility of our observations and suggests that the PM‐MRI findings do not simply reflect nonspecific postmortem artifact.

In contrast, non‐SUDEP epilepsy cases in the present cohort exhibited more pronounced volume reductions relative to healthy in vivo controls. This divergence argues against the interpretation that the observed volumetric differences in SUDEP reflect chronic epilepsy‐related neurodegeneration alone. Instead, relative volume preservation or enlargement in SUDEP cases is consistent with transient peri‐ictal or post‐ictal processes, such as seizure‐related intracellular edema, which have been well described on MRI following generalized tonic–clonic seizures. Post‐ictal imaging changes have been reported in limbic and subcortical regions involved in autonomic and respiratory regulation, overlapping with structures repeatedly implicated in SUDEP vulnerability.^3^, ^4^, ^6^

These findings support the interpretation that PM‐MRI captures a structural “snapshot” of peri‐ or post‐ictal brain states at the time of death, rather than fixed lesions that directly cause SUDEP. In this sense, our data align with the prevailing concept that SUDEP is fundamentally a post‐ictal event, occurring during a period of impaired autonomic, respiratory, and arousal recovery, rather than the consequence of a static structural abnormality.^1^, ^15^ Importantly, we do not claim that the observed edema or volumetric changes are themselves causal. Rather, they likely represent imaging correlates of severe terminal or near‐terminal seizures, which may distinguish SUDEP from deaths occurring after earlier, non‐terminal seizures on the same day.

The observation that volumetric differences extend beyond classical SUDEP‐associated regions (e.g., hippocampus and amygdala) to include thalamus and basal ganglia suggests a more global effect, consistent with widespread seizure‐related metabolic and cellular disturbances. This systematic pattern supports the view that the findings are not regionally specific and cautions against overinterpretation of mechanistic pathways. It also raises the possibility that non‐SUDEP cases, both of whom died following prolonged systemic illness, may have experienced additional factors such as hypoxia, inflammation, or prolonged intensive care that contributed to reduced volumes at the time of death.

Our structural findings should be interpreted in the context of emerging non‐imaging biomarkers of SUDEP. Recent work by Mangana‐Téllez and colleagues in *The Lancet Neurology* identified EEG‐based markers of impaired post‐ictal recovery and autonomic instability that prospectively predict SUDEP risk.^8^ Together, these EEG findings and the present PM‐MRI data converge on a shared biological theme: SUDEP risk is characterized less by fixed anatomical lesions and more by impaired post‐ictal brain–body integration, affecting arousal, respiration, and cardiovascular control. Structural imaging may therefore provide complementary information by identifying brain regions that are particularly vulnerable to these peri‐ictal disturbances.

Notably, large population‐based and prospective studies continue to demonstrate that, beyond frequent generalized tonic–clonic seizures and sleeping alone, few validated clinical or biological risk biomarkers for SUDEP currently exist.^10^, ^11^, ^14^ The convergence of in vivo MRI, PM‐MRI, and EEG‐based biomarkers suggests that multimodal approaches will be required to bridge the gap between epidemiological risk factors and underlying pathophysiology.

This study has several important limitations. The sample size is small, postmortem intervals were variable, and complete neuropathological examinations were not performed. PM MRI signal properties evolve with time after death, leading to blurring of gray–white matter boundaries which could show as gray matter volume loss. Nevertheless, the persistence of detectable volumetric differences days after death, even without controlling for all postmortem confounders, is striking and supports the feasibility of PM‐MRI as a tool for SUDEP research. Importantly, our findings should be viewed as hypothesis‐generating and descriptive, rather than mechanistic.

Despite these limitations, PM‐MRI offers a unique and underutilized opportunity to investigate SUDEP. Unlike ante‐mortem imaging, it allows direct linkage between quantitative MRI measures and histopathology within the same brain, enabling validation of imaging biomarkers against cellular and molecular substrates.^16^ By integrating PM MRI with histology and, where available, ante‐mortem imaging and electrophysiology, future studies may clarify which imaging signatures reflect transient peri‐ictal states and which represent enduring vulnerabilities.

In summary, our findings demonstrate that PM‐MRI can identify consistent structural differences in SUDEP cases that closely parallel in vivo observations and emerging EEG biomarkers. While not providing a mechanistic explanation for death, PM‐MRI represents a promising complementary approach for identifying and validating SUDEP‐related biomarkers, with potential implications for risk stratification, prevention strategies, and the interpretation of negative conventional autopsy findings.

## AUTHOR CONTRIBUTIONS

Conceptualization: AH, MT, OJA, DM, and MJK. Methodology (MRI acquisition and analysis): AH, FX, HB, OJA, DM, and MJK. PM‐MRI and scalpel MRI Investigation: AH, DM, MT, HB, OJA, and MJK. Image processing, software, and quantitative MRI analyses: F.X. (lead), with contributions from BD and MK. Writing—original draft: A.H., F.X., M.K. Writing—review and editing: all authors. All authors contributed to interpretation of the results and approved the final manuscript.

## FUNDING INFORMATION

This work was undertaken in part at University College London (UCL)/UCLHospital, which receives support from the National Institute for Health Research (NIHR) UCL Hospitals Biomedical Research Centre. This study was funded by the Wellcome Trust (221 934/Z/20/Z); F.X. received direct support from this grant, MT and MJK were the grant holders. The Epilepsy Society supports AH, HB and the Brain and Tissue Bank at UCL. This study was supported by the NIHR University College London Hospitals Biomedical Research Centre (BRC540/HEI/EA/110410). J.S.D. was supported by the National Institute for Health and Care Research (NIHR) and the Wellcome Trust (218380). The funders had no role in the design and conduct of the study; collection, management, analysis, and interpretation of the data; preparation, review, or approval of the manuscript; and decision to submit the manuscript for publication.

## CONFLICT OF INTEREST STATEMENT

The authors have nothing to report. We confirm that we have read the Journal’s position on issues involved in ethical publication and affirm that this report is consistent with those guidelines.

## Data Availability

The data that support the findings of this study are available on request from the corresponding author. The data are not publicly available due to privacy or ethical restrictions.
